# Pre-Activation Dynamics of Category-Specific Attentional Templates in Visual Search

**DOI:** 10.3390/bs15121606

**Published:** 2025-11-21

**Authors:** Jiahui Su, Xin Ling, Zhiwei Miao, Rongtao Wu, Yunpeng Jiang, Xia Wu

**Affiliations:** 1 Key Research Base of Humanities and Social Sciences of the Ministry of Education, Academy of Psychology and Behavior, Tianjin Normal University, Tianjin 300387, China; ssucog@gmail.com (J.S.); jiangyp@tjnu.edu.cn (Y.J.); 2 Faculty of Psychology, Tianjin Normal University, Tianjin 300387, China; 3 School of Psychology, South China Normal University, Guangzhou 510631, China; 4 Department of Psychology, Zhejiang Normal University, Jinhua 321004, China; 5 Tianjin Key Laboratory of Student Mental Health and Intelligence Assessment, Tianjin 300387, China

**Keywords:** category, attentional templates, experience, learning, MVPA, visual search

## Abstract

Visual search can be guided and facilitated by a category-specific attentional template (CAT) when searching for a target embedded into a set of categories. While previous research has proven that CATs can be pre-activated, it has caused confusion due to inconsistent task difficulty and unbalanced target-defined dimensions. To avoid the unbalanced dimensions and investigate the effects of category frameworks on the time course of the pre-activation of CAT, we defined the targets as a color-defined category based on prototype (warm, cool), semantic (garden, ocean), and strategy (red–blue, yellow–green). Moreover, to maintain the consistency of task difficulty, we employed the identification task to all frameworks of CAT within the RSPP paradigm and tracked the time course of CAT pre-activation by measuring N2pcs elicited by the probes sequentially occurring prior to the target. ERP and MVPA results found that the semantic-based CAT (involving in experience and learning) was pre-activated first, approximately 1200 ms before the search display, followed by the prototype-based CAT (involving in perceptual similarity) at about 900 ms, and finally the strategy-based CAT (involving in learning) at around 600 ms before the search display. By balancing the target dimensions and maintaining the difficulty of tasks, the findings provide further evidence for differences in the activation patterns of various constitutive CATs.

## 1. Introduction

In complex information environments, an attentional template (AT), referring to a mental representation stored in working memory, can effectively guide attentional selection (Duncan & Humphreys, 1989; Vickery et al., 2005). Relative to simple features such as color and shape, a category that combines multiple objects into a specific set is more likely to be chosen as a target in daily life (e.g., searching for a food category rather than a yellow feature when hungry). When searching for a target within a category, a category-specific attentional template (CAT) is generated to improve recognition performance for that specific category (Zivony & Eimer, 2022). Previous research has shown that CATs can be pre-activated to facilitate target identification (Lewis-Peacock et al., 2015; Lim et al., 2021; Miao et al., 2023). This pre-activation occurs before target onset and is distinct from the activation evoked by target processing. Given that categories play a more significant role than single features in daily life, investigating CAT is essential for understanding how individuals modulate attention during visual searches in everyday life.

CAT can be tuned to different category frameworks. Category frameworks are defined as prototype-based or semantic-based, depending on different patterns of configuration (Miao et al., 2023). On the one hand, a prototype refers to a generalized and central description of commonalities among items within a category, and it can be categorized based on perceptual similarity (Smith & Minda, 1998). For instance, when searching for the apple category, the objects within the category are grouped by their similarities with the prototypical features, such as red color and circular shape. Such a prototype-based category can reduce the need to remember the specific instances within the category (Yeh & Peelen, 2022). Importantly, a prototype-based CAT has been shown to play an active role in guiding the search for objects defined by prototypical features (Grubert & Eimer, 2018; Miao et al., 2023). On the other hand, semantic frameworks generalize from high-level conceptual information and represent linguistic connections among objects within a category (Inhoff et al., 2018; Miao et al., 2023). For instance, the concept of a “garden” can serve as a semantic representation associated with green leaves and relaxing experiences, rather than fixed common features. When searching for objects defined by such a conceptual representation, a semantic-based CAT has been shown to be generated to facilitate the identification of targets within a category (Miao et al., 2023). However, the semantic representation is influenced by both learning and experience (Pecher & Zeelenberg, 2018; Rehrig et al., 2021). Experience can be seen as knowledge obtained from objective phenomena, while learning refers to information recently obtained through practice. Although both experience and learning can facilitate attentional selection in visual search (Addleman & Jiang, 2019; Brockmole & Le-Hoa Võ, 2010; Rehrig et al., 2021; Võ & Wolfe, 2012), the separate effects on CAT still need to be distinguished.

Miao et al. (2023) investigated the time course of CAT pre-activation under different category frameworks, and the results showed that the prototype-based CAT exhibited a later and smaller pre-activation relative to the semantic-based CAT. However, variations in task difficulty and target-defined dimensions introduced some confusion in this study. Firstly, in their experimental design, an identification task (requiring participants to report the target’s orientation) was used for the prototype-based category, whereas an easier detection task (reporting the target’s location) was employed for the semantic-based category. This difference implies that the observed effects cannot be solely attributed to the category itself. Specifically, the delayed pre-activation observed in the prototype-based CAT can be explained by the higher attentional demands of the more challenging identification task, which led to insufficient attentional resources during the pre-activation process (Huang & Pashler, 2005; Volosin & Horváth, 2020). Secondly, they defined the prototype-based category by the colors of “warm” and “cool”, while defining the semantic-based category by the shapes of “letter” and “digital”. Therefore, the smaller pre-activation of prototype-based CAT can be explained by the fact that the color-defined targets in the prototype framework require fewer attentional resources than the shape-defined ones (Barras & Kerzel, 2017; Eimer, 1996; Wu et al., 2020). In these cases, balancing the task difficulty and target dimensions across different frameworks of CAT is necessary to avoid the above-highlighted confusion and clarify the time course of different CATs.

The aim of the present study was to investigate the effects of category frameworks on the time course of CAT pre-activation. Firstly, to isolate the learning effect from semantic-based CAT, we added a strategy-based category that purely combines objects into a category by learning rather than experience, keeping to the three conditions of prototype-based, semantic-based, and strategy-based category as targets. Secondly, to ensure the consistency of target-defined dimensions, we defined all frameworks of the categories by a unified color dimension. Thirdly, to unify the task difficulty across all frameworks, participants were required to respond to the orientation of the target in all conditions. According to the hierarchy theory (Hochstein & Ahissar, 2002), prototype-based CATs rely on low-level perceptual features, allowing stimuli within the category to be processed rapidly, whereas semantic-based CATs depend on high-level conceptual representations, which are processed more slowly. Therefore, we hypothesize that prototype-based CATs require less preparation time, which would be reflected by later pre-activation, whereas semantic-based CATs require more preparation time, which would be reflected by earlier pre-activation.

To test these hypotheses and track the time course of CAT pre-activation, we employed the rapid serial probe presentation (RSPP) paradigm presented by Grubert (Grubert & Eimer, 2018). In this paradigm, participants are presented with a series of color singleton probes that are either matched or mismatched with the target feature. Each probe appears briefly before the onset of the search display, allowing the measurement of transient neural responses to each probe. By measuring the N2pc (a negativity component in posterior scalp electrodes contralateral to the candidate target that reflects the attentional allocation (Luck & Hillyard, 1994)) and P_D_ components (a positivity component in scalp electrodes posterior to the distractor that reflects the attentional suppression (Hickey et al., 2006)), elicited by each probe, we examined the time course of CAT pre-activation. This design allows for a clearer examination of how different CATs are pre-activated over time, addressing limitations in previous studies that confounded task difficulty or target dimensions. To minimize the potential overlap of ERP components (e.g., N2pc or Pd) between adjacent probes, each probe was followed by a blank interval, and both the probe and blank durations were extended relative to the original paradigm to ensure the clearer temporal separation of neural responses. Based on prior evidence using the same paradigm (Miao et al., 2023), six probe–blank cycles were employed, as this number provides the earliest reliable tracking of pre-activation patterns while maintaining a clear temporal resolution across the pre-search period.

## 2. Methods

### 2.1. Participants

An a priori power analysis, conducted using More Power 6.0.4 (Campbell & Thompson, 2012), indicated that a repeated-measures ANOVA of 3 × 2 × 6 with the factors of category framework (prototype, semantic, and strategy), matching degree (match vs. mismatch), and probe (1–6) required a sample of at least 30 participants (an effect size of 0.15 represents eta squared, an alpha error of 0.05, and a 0.80 statistical power). Initially, thirty-three participants took part in the study. However, one participant was excluded due to an accuracy rate below 50%, and two others were excluded due to the ERP-rejected trials exceeding 33%. Statistical analysis was performed on the remaining 30 participants (*M* = 20.23 years; *SD* = 2.07 years; 19 females). This study was approved by the Ethics Committee. All participants provided written consent prior to testing and reported normal or corrected-to-normal vision.

### 2.2. Materials and Procedure

**Figure 1:**
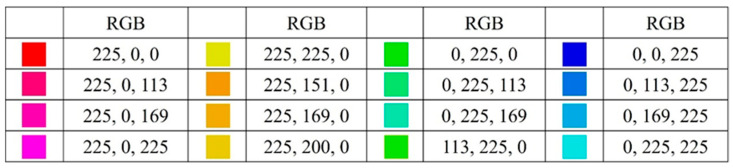
Experimental material of stimulus. Prototype-based category included warm and cool color, the warm color composed of the first and second columns, and the cool color composed of the third and fourth columns. Semantic-based category included garden and ocean color, the garden color composed of the first and third columns and the ocean color composed of the second and fourth columns. Strategy-based category included red–blue and yellow–green color, the red–blue color composed of the first and fourth columns and the yellow–green color composed of the second and third columns.

The experiment was presented on a 16-inch CRT monitor (1024 × 768 pixels, 60 Hz refresh rate) at a viewing distance of approximately 60 cm, controlled by a Windows 10 PC. Stimulus presentation and response collection were managed using the E-Prime software (version 3.0). Color stimuli RGB values were adapted from Miao et al. (2023), with adjustments to maintain consistent brightness and saturation across stimuli. As shown in Figure 1, 16 colors were grouped into four color systems: red, yellow, green, and blue. In the prototype-based category framework, categories were defined as warm (red and yellow) versus cool (green and blue). In the semantic-based category framework, categories were defined as garden (red and green) versus ocean (yellow and blue). In the strategy-based category framework, categories were arbitrarily defined as red–blue and yellow–green. Within each framework, one category was assigned as the target and the other as the non-target.

**Figure 2:**
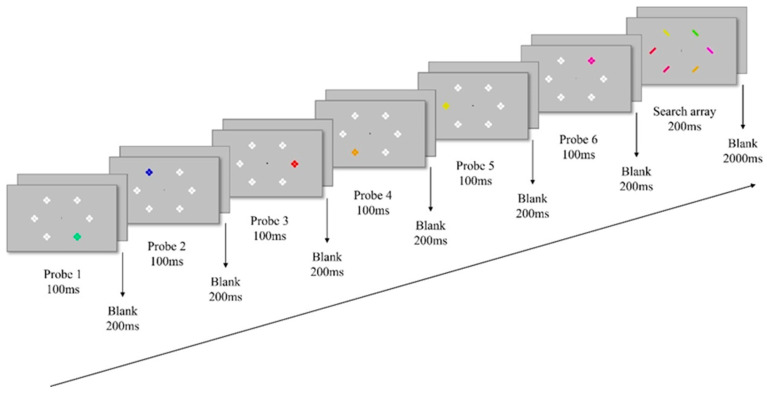
Schematic representation of the procedure. Participants were instructed to indicate whether the target was tilted to left or right, while the target color was determined by its category. In this particular case, the target was the green rectangle tilted to left chosen from the cool color category. Before the search task, participants were shown six sequential probes, where the color of the critical probes was randomly selected to match the target-defining category (matching) or the non-target-defining category (mismatching).

As shown in Figure 2, the experiment employed an RSPP paradigm. Each trial began with six consecutive probe displays, each presented for 100 ms and followed by a 200 ms blank, prior to the search display. Each probe display included a single task-relevant colored probe stimulus and five task-irrelevant white stimuli, arranged equidistantly around a central fixation cross (1° × 1°). Each probe stimulus (0.5° × 0.5°) was composed of two vertical and two horizontal dots (0.1° × 0.1°) and appeared randomly at one of the six locations. The color of the probe stimulus was randomly drawn either from the target category (matching condition) or the non-target category (mismatching condition) within the current category framework. Participants were instructed that the probe displays were task-irrelevant and required no response.

Following the probe sequence, a search display was presented for 200 ms, consisting of six colored rectangles (1° × 1°), each tilted either left or right. The target was defined using the same principle as the probe displays; its color belonged to the target category of the current category framework (e.g., a cool color in the prototype-based framework). Following the search display, a 2000 ms blank screen was presented, during which participants indicated the orientation (left or right) of the target rectangle via keyboard as quickly and accurately as possible.

Before the main experiment, participants were required to complete a category learning phase, where they were instructed to memorize the color–category associations for each framework. For example, in the semantic condition, they were required to color drawings representing “garden” and “ocean” themes using crayons of the corresponding category colors (e.g., red flowers and green leaves for “garden”). After this, participants completed a 48-trial classification task, where they categorized single colors into their correct subcategories. Only participants who achieved 100% accuracy were allowed to proceed to the main task. The formal experiment began with 12 practice trials, followed by 6 blocks of 100 trials each (corresponding to warm, cool, garden, ocean, red–blue, and yellow–green categories, presented in randomized order), totaling 612 trials. The order of the three category frameworks was randomized across participants, and within each framework, the two corresponding blocks were presented consecutively. Participants were allowed to rest between blocks, and the entire session lasted approximately 40 min.

### 2.3. EEG Recording and Analysis

Electroencephalographic (EEG) data were recorded using an extended International 10–20 with 64 channels (Neuroscan Inc., Charlotte, NC, USA). Impedances were kept below 5 kΩ, and Ref, which is located in the central district, adjacent to Cz, was used as the reference electrode during recording. Offline EEG analysis was conducted using the EEGLAB 2023.1 toolbox (Delorme & Makeig, 2004) in MATLAB 2021b and custom-written MATLAB functions. Each scalp electrode was referenced to the average of M1 and M2. The raw data were subjected to a 0.1 Hz high-pass and a 30 Hz low-pass filter. Subsequently, all signals were resampled offline at 500 Hz. The filtered data were segmented into epochs ranging from −200 ms to 400 ms relative to stimulus onset. Baseline correction was used to reduce the task deviation. Independent component analysis (ICA) was employed to reject blinks and eye movement artifacts (Delorme & Makeig, 2004; Mognon et al., 2011). EEG segments with voltages exceeding ±100 μV were excluded from analysis, with an average rejection rate of 4.64% (range: 0.02–15.11%).

The waves elicited by 13 conditions of 6 (probe: 1–6) × 2 (matching degree: match vs. mismatch) and the search display for each category framework were averaged. Contralateral waveforms were computed as the average of the PO7 electrode for right-sided stimuli and the PO8 electrode for left-sided stimuli. The ipsilateral waveforms were computed as the average of the PO7 electrode for left-sided stimuli and the PO8 electrode for right-sided stimuli. Different waveforms were computed by subtracting the ipsilateral from the contralateral waveform. The N2pc and P_D_ time window were based on the significant difference between contralateral and ipsilateral waveforms, calculated using cluster-based permutation (*p* < 0.05) (Maris & Oostenveld, 2007). The time window for computing the mean amplitude of N2pc was 240–280 ms for the search display, 180–260 ms for probes in match condition, and 130–210 ms for probes in the mismatch condition. The time window for P_D_ was 90–170 ms for probes in the mismatch condition. Multiple comparisons were controlled by setting the significance threshold for false discovery rate (FDR) (Storey, 2002) to 0.1 (Tung et al., 2021). Unless otherwise noted, tests had an FDR q-value lower than or equal to 0.1 in this study.

### 2.4. Multivariate Pattern Analysis

A supervised learning classification approach was used to further investigate differences among category frameworks in the time-resolved EEG. We used a support vector machine (SVM), as implemented in the MVPA-Light toolbox for MATLAB (Treder, 2020), to perform the multiclass classification for the data of each participant before 2000 ms and after 100 ms of search display. EEG preprocessing was approximately consistent with ERP analysis but resampled at 50 Hz, hence a 20 ms resolution. To ensure sufficient trial for SVM training, we did not reject the extremum voltages. Decoding analyses were conducted using the distributed amplitudes of the raw EEG signal over each electrode and time point. Using 60 electrodes thus resulted in 60 features for three classes of whole brain pre-active mode of category frameworks. Each participant’s dataset was analyzed using a 5-fold cross-validation training-testing scheme. In this scheme, the data was segmented into 5 equally sized folds (each fold containing a near-equal number of trials, with equal distributions of the three category frameworks across the folds). The classifier was trained on 80% of the data (4 of the 5 folds), learning to distinguish between the different stimulus classes. The validity of the trained classifier was tested on the remaining 20% of the data. All data was tested once by repeating the procedure 5 times without ever using the same data for training and for testing. Classification performance was evaluated using the traditional accuracy (ACC) metric, defined as the percentage of correct predictions.

## 3. Results

### 3.1. Behavioral Results

**Figure 3:**
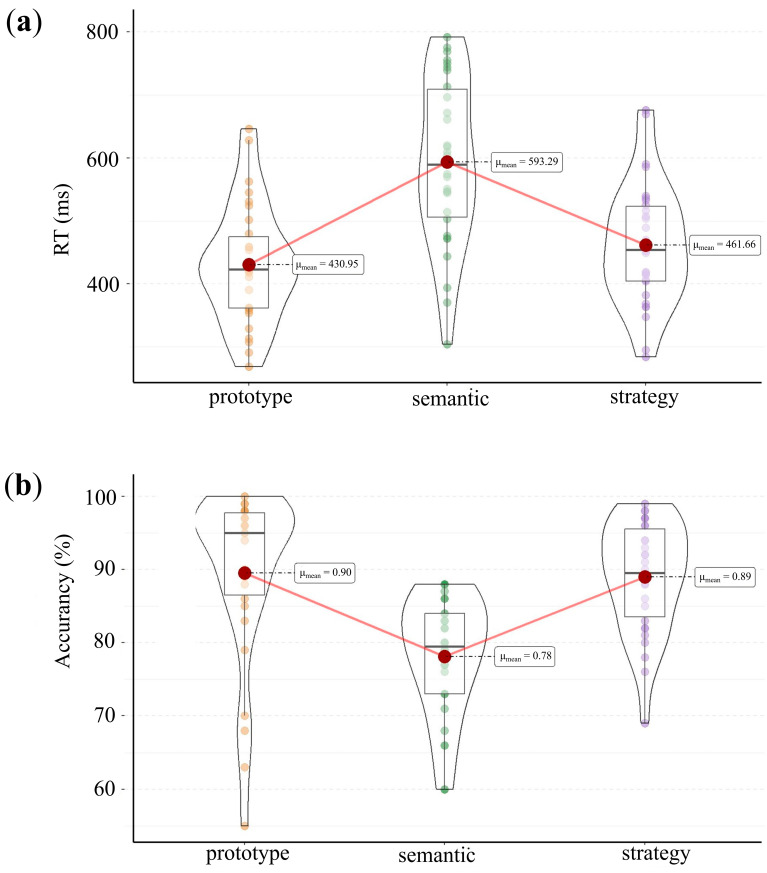
(**a**) RT and (**b**) accuracy results for each category framework. Horizontal bars within boxes denote medians. Tops and bottoms of boxes represent 25th and 75th percentiles, and lines extend to the 1.5× interquartile range.

For each participant, we excluded the incorrect responses and reaction times (RTs) exceeding 2.5 SDs. To examine the differences in target identification across the three category frameworks, we conducted repeated-measures ANOVAs on the accuracy and RTs for each framework (prototype, semantic and strategy) (Figure 3).

The accuracy and RTs of each framework were analyzed within two separate one-way repeated measures ANOVAs. For accuracy, a main effect of category framework was seen [*F* (2,58) = 43.23, *p* < 0.001, *η_p_*^2^ = 0.76]. Accuracy in the semantic condition (*M* = 78.10%, *SD* = 8.10%) was significantly lower than in both the prototype (*M* = 89.53%, *SD* = 11.67%, *p* < 0.001) and strategy (*M* = 88.97%, *SD* = 7.51%, *p* < 0.001) conditions. For RTs, a significant main effect of category framework was observed [*F* (2,58) = 42.80, *p* < 0.001, *η_p_*^2^ = 0.75]. RTs in the prototype (*M* = 430.95 ms, *SD* = 93.25 ms, *p* < 0.001) and strategy (*M* = 461.66 ms, *SD* = 96.53 ms, *p* < 0.001) conditions were significantly shorter than those in the semantic condition (*M* = 593.29 ms, *SD* = 130.89 ms). Furthermore, RTs in the prototype condition were significantly shorter than those in the strategy condition (*p* = 0.027).

### 3.2. ERP Results

#### 3.2.1. N2pcs of Search Display Under Different Category Frameworks

We first confirmed that there were differences among the three category frameworks on the search display. ERPs were submitted to a 3 × 2 repeated-measures ANOVA with the factors of category framework (prototype, semantic, strategy) and laterality (contralateral vs. ipsilateral). Results showed that there was no main effect of category framework [*F* (2,58) = 0.37, *p* = 0.691], but a significant main effect of laterality [*F* (1,29) = 167.46, *p* < 0.001, *η_p_*^2^ = 0.75], indicating a more negative amplitude of contralateral (*M* = −0.44 μV, *SE* = 0.83) compared to ipsilateral (*M* = 1.49 μV, *SE* = 0.81). In addition, there was a significant interaction of category framework × laterality [*F* (2,58) = 8.45, *p* < 0.001, *η_p_*^2^ = 0.27], showing that the N2pc differed across the three frameworks (*p*s < 0.001).

**Figure 4:**
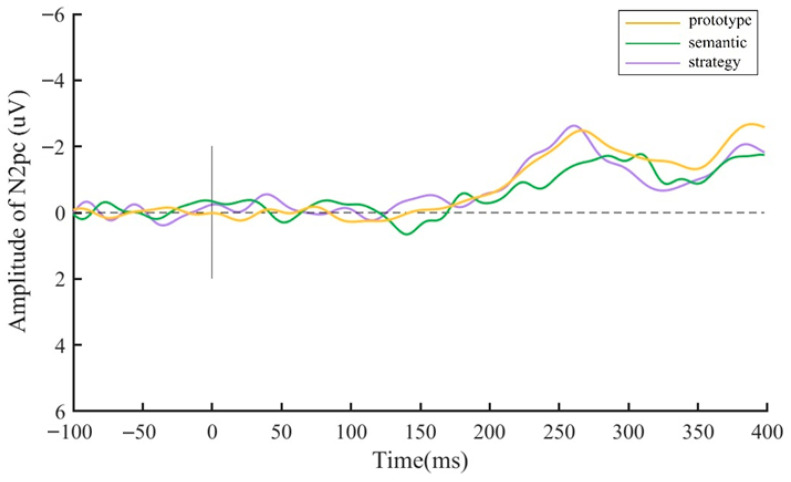
Difference waves when searching in the search displays under three categories.

To further examine this effect, a one-way repeat-measures ANOVA conducted on N2pcs revealed a significant main effect of category framework [*F* (2,58) = 8.45, *p* < 0.001, *η_p_*^2^ = 0.23]. Figure 4 shows that the N2pc elicited in the prototype (*M* = −2.22 μV, *SE* = 0.28, *p* = 0.005) and strategy conditions (*M* = −2.26 μV, *SE* = 0.28, *p* = 0.001) was significantly larger than that in the semantic condition (*M* = −1.31 μV, *SE* = 0.20).

#### 3.2.2. N2pcs of Probes Under Different Category Frameworks

To examine the difference among three category frameworks across probes, the ERP mean amplitudes of each probe display were firstly submitted to a 3 × 2 × 2 × 6 repeated-measures ANOVA with the factors of category framework (prototype, semantic, strategy), matching degree (match vs. mismatch), laterality (contralateral vs. ipsilateral), and probe (1–6). The ANOVA results indicated a significant main effect of category framework [*F* (2,58) = 5.00, *p* = 0.010, *η_p_*^2^ = 0.15], showing that the mean amplitudes elicited in the prototype (*M* = −1.85 μV, *SE* = 0.25) and semantic conditions (*M* = −1.79 μV, *SE* = 0.27) were larger than in the strategy condition (*M* = −1.41 μV, *SE* = 0.22, *p* = 0.018, *p* = 0.014, respectively). There was a significant main effect of probe [*F* (5,145) = 8.60, *p* < 0.001, *η_p_*^2^ = 0.23], showing that the mean amplitudes shown at probe 1 (*M* = −2.62 μV, *SE* = 0.48), probe 2 (*M* = −2.65 μV, *SE* = 0.39), and probe 6 (*M* = −2.01 μV, *SE* = 0.37) were larger than those shown at probe 3 (*M* = −0.98 μV, *SE* = 0.38, *p*s ≤ 0.043) and probe 4 (*M* = −0.20 μV, *SE* = 0.36, *p*s < 0.001). The mean amplitude at probe 2 was larger than that at probe 5 (*M* = −1.60 μV, *SE* = 0.26, *p* = 0.041), and the mean amplitude at probe 5 was larger than those at probe 4 (*p* = 0.001).

Although no main effect of matching degree [*F* (1,29) = 0.18, *p* = 0.678] or laterality [*F* (1,29) = 1.12, *p* = 0.298] were found, a significant matching degree × laterality interaction [*F* (1,29) = 12.95, *p* = 0.001, *η_p_*^2^ = 0.31] showed that reliable N2pcs were elicited by target-matched stimuli [*F* (1,29) = 6.68, *p* = 0.015, *η_p_*^2^ = 0.19], whereas reliable P_D_s were elicited by target-mismatched stimuli [*F* (1,29) = 5.71, *p* = 0.024, *η_p_*^2^ = 0.17]. In addition, a significant interaction of matching degree × probe was found [*F* (5,145) = 7.03, *p* < 0.001, *η_p_*^2^ = 0.20], showing that amplitudes elicited by target-matched stimuli were larger than those elicited by target-mismatched stimuli at probe 3 [*F* (1,29) = 12.12, *p* = 0.002, *η_p_*^2^ = 0.30], probe 4 [*F* (1,29) = 17.01, *p* < 0.001, *η_p_*^2^ = 0.37], and probe 6 [*F* (1,29) = 4.65, *p* = 0.040, *η_p_*^2^ = 0.14]. A category × probe interaction [*F* (10,290) = 6.23, *p* < 0.001, *η_p_*^2^ = 0.18] showed that amplitudes in the prototype condition were significantly larger than those in the semantic and strategy conditions at probe 1 [*F* (2,28) = 11.44, *p* < 0.001, *η_p_*^2^ = 0.45] and probe 6 [*F* (2,28) = 3.61, *p* = 0.040, *η_p_*^2^ = 0.21]. At probe 2, amplitudes in the prototype and strategy conditions were larger than those in the semantic condition [*F* (2,28) = 4.94, *p* = 0.015, *η_p_*^2^ = 0.26], whereas at probe 4, amplitudes in the semantic condition were larger than those in the prototype and strategy conditions [*F* (2,28) = 9.93, *p* < 0.001, *η_p_*^2^ = 0.42].

There was a significant three-way interaction of category framework × matching degree × probe [*F* (10,290) = 3.70, *p* < 0.001, *η_p_*^2^ = 0.11]. A three-way interaction between matching degree × laterality × probe was also observed [*F* (5,145) = 3.90, *p* = 0.002, *η_p_*^2^ = 0.12]. However, the four-way interaction of category framework × matching degree × laterality × probe was not significant [*F* (10,290) = 0.75, *p* = 0.67], possibly due to lateralization effects occurring across conditions.

**Figure 5:**
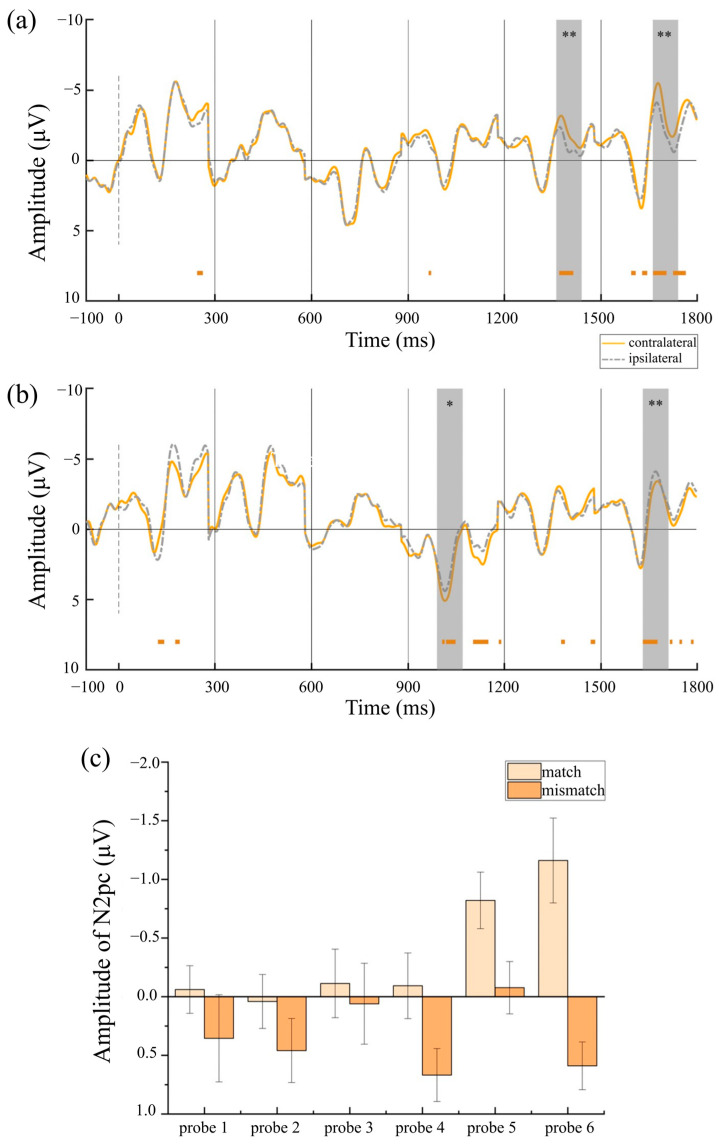
ERP results in the prototype condition. (**a**) ERPs of probe 1–6 in match condition. (**b**) ERPs of probe 1–6 in mismatch condition. (**c**) Prototype-based CAT pre-activation. The error bars indicate the standard errors. The cluster-based permutation test identifies significant clusters with negative effects by displaying colored bars below the waveform (1000 permutations, alpha level of 0.05). * *p* < 0.05, and ** *p* < 0.01.

**Figure 6:**
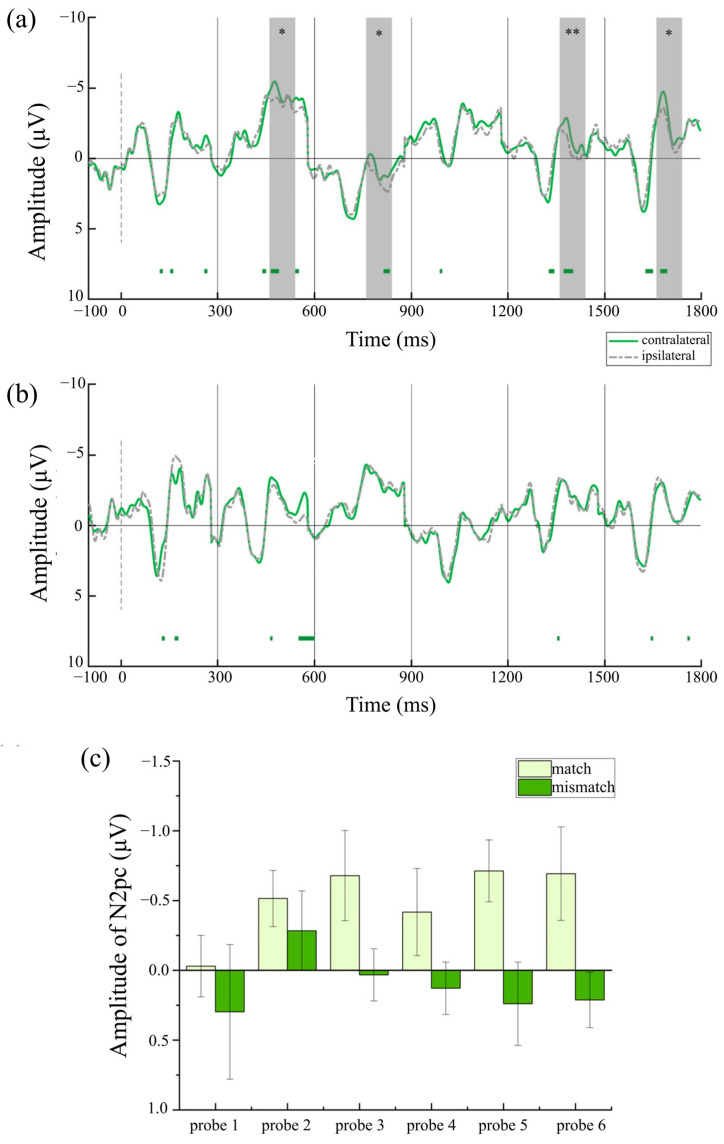
ERP results in the semantic condition. (**a**) ERPs of probe 1–6 in match condition. (**b**) ERPs of probe 1–6 in mismatch condition. (**c**) Semantic-based CAT pre-activation. The error bars indicate the standard errors. The cluster-based permutation test identifies significant clusters with negative effects by displaying colored bars below the waveform (1000 permutations, alpha level of 0.05). * *p* < 0.05, and ** *p* < 0.01.

**Figure 7:**
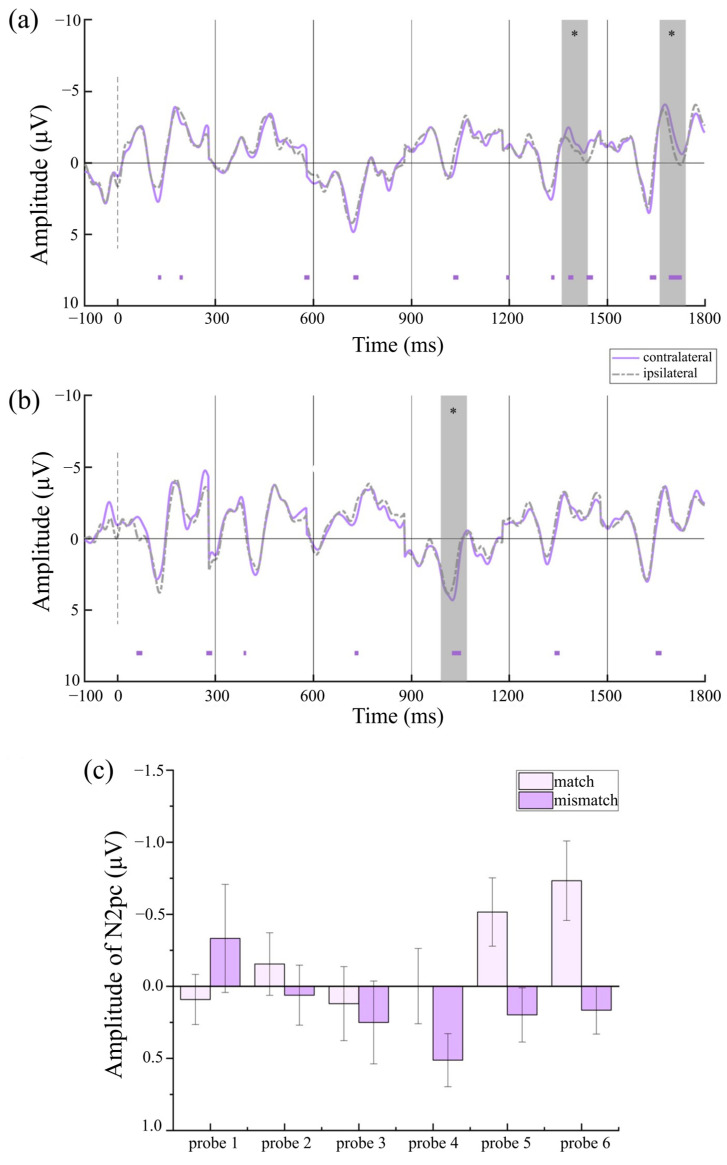
ERP results in the strategy condition. (**a**) ERPs of probe 1–6 in match condition. (**b**) ERPs of probe 1–6 in mismatch condition. (**c**) Strategy -based CAT pre-activation. The error bars indicate the standard errors. The cluster-based permutation test identifies significant clusters with negative effects by displaying colored bars below the waveform (1000 permutations, alpha level of 0.05). * *p* < 0.05.

To investigate whether reliable lateralized components were elicited in each condition, paired-sample *t*-tests were conducted to compare contralateral and ipsilateral amplitudes. Under the prototype-based category framework, Figure 5a shows reliable N2pcs elicited by target-matched stimuli at probe 5 (*t* (29) = −3.41, *p* = 0.002, Cohen’s *d* = −0.62, 95% CI = [−1.01, −0.23]) and probe 6 (*t* (29) = −3.21, *p* = 0.003, Cohen’s *d* = −0.59, 95% CI = [−0.97, −0.19]), whereas Figure 5b shows reliable P_D_s elicited by target-mismatched stimuli at probe 4 (*t* (29) = 2.95, *p* = 0.006, Cohen’s *d* = 0.54, 95% CI = [0.15, 0.92]) and probe 6 (*t* (29) = 2.88, *p* = 0.007, Cohen’s *d* = 0.53, 95% CI = [0.14, 0.90]). Under the semantic-based category framework, Figure 6a shows reliable N2pcs elicited by target-matched stimuli at probe2 (*t* (29) = −2.57, *p* = 0.016, Cohen’s *d* = 0.47, 95% CI = [−0.84, −0.09]), probe 3 (*t* (29) = −2.11, *p* = 0.044, Cohen’s *d* = 0.38, 95% CI = [−0.75, −0.01]), probe 5 (*t* (29) = −3.34, *p* = 0.003, Cohen’s *d* = 0.59, 95% CI = [−0.97, −0.19]), and probe 6 (*t* (29) = −2.07, *p* = 0.048, Cohen’s *d* = 0.38, 95% CI = [−0.75, 0.00]), and Figure 6b shows that there was no N2pc elicited by target-mismatched stimuli at any probe. Under the strategy-based category framework, Figure 7a shows reliable N2pcs elicited by target-matched stimuli at probe 5 (*t* (29) = −2.18, *p* = 0.038, Cohen’s *d* = −0.40, 95% CI = [−0.77, −0.02]) and probe 6 (*t* (29) = −2.66, *p* = 0.013, Cohen’s *d* = −0.49, 95% CI = [−0.86, −0.10]), whereas Figure 7b also shows reliable P_D_s elicited by target-mismatched stimuli at probe 4 (*t* (29) = 2.77, *p* = 0.010, Cohen’s *d* = 0.51, 95% CI = [0.12, 0.88]).

To explore the time course and strength of CAT pre-activation with different frameworks, the different waveforms were submitted to a repeated-measures ANOVA of 2 × 6 with the factors of matching degree (match vs. mismatch) and probe (1–6) for each category framework. In the prototype condition, there was a significant main effect of matching degree [*F* (1,29) = 10.98, *p* = 0.002, *η_p_*^2^ = 0.28], showing larger amplitudes of target-matched stimuli (*M* = −0.37 μV, *SE* = 0.17) than those elicited by target-mismatched stimuli (*M* = 0.34 μV, *SE* = 0.13). There was also a main effect of probe [*F* (5,145) = 2.60, *p* = 0.028, *η_p_*^2^ = 0.08]. The N2pcs at probe 5 (*M* = −0.45 μV, *SE* = 0.18) were larger than those at probe 1 (*M* = 0.15 μV, *SE* = 0.20, *p* = 0.052), probe 2 (*M* = 0.25 μV, *SE* = 0.15, *p* < 0.001), and probe 4 (*M* = 0.29 μV, *SE* = 0.21, *p* = 0.003), while the N2pcs at probe 6 (*M* = −0.29 μV, *SE* = 0.21) were significantly larger than those at probe 2 (*p* = 0.018) and probe 4 (*p* < 0.001). There was a significant matching × probe interaction [*F* (5,145) = 2.50, *p* = 0.033, *η_p_*^2^ = 0.08]. Figure 5c shows there were larger amplitudes elicited by target-matched stimuli than those elicited by target-mismatched stimuli at probe 4 [*F* (1,29) = 6.32, *p* = 0.018, *η_p_*^2^ = 0.18], probe 5 [*F* (1,29) = 6.93, *p* = 0.013, *η_p_*^2^ = 0.19] and probe 6 [*F* (1,29) = 17.72, *p* < 0.001, *η_p_*^2^ = 0.38]. For target-matched stimuli, the amplitude of the N2pcs at probe 5 (*M* = −0.82 μV, *SE* = 0.24) and probe 6 (*M* = −1.16 μV, *SE* = 0.36) were larger than at probe 1 (*M* = −0.06 μV, *SE* = 0.20, *p* = 0.023, *p* = 0.009, respectively), probe 2 (*M* = 0.041 μV, *SE* = 0.23, *p* = 0.009, *p* = 0.002, respectively), probe 3 (*M* = −0.11 μV, *SE* = 0.29, *p* = 0.015, *p* = 0.010, respectively), and probe 4 (*M* = −0.09 μV, *SE* = 0.28, *p* = 0.019, *p* < 0.001, respectively). For target-mismatched stimuli, P_D_ amplitudes at probe 2 (*M* = 0.46 μV, *SE* = 0.27), probe 4 (*M* = 0.67 μV, *SE* = 0.23), and probe 6 (*M* = 0.59 μV, *SE* = 0.20) were larger than at probe 5 (*M* = −0.08 μV, *SE* = 0.22, *p* = 0.080, FDR q-value = 0.151, *p* = 0.013, *p* = 0.022, respectively).

In the semantic condition, a main effect of matching degree was observed [*F* (1,29) = 10.34, *p* = 0.003, *η_p_*^2^ = 0.26], with larger amplitudes for target-matched stimuli (*M* = −0.51 μV, *SE* = 0.17) than elicited by target-mismatched stimuli (*M* = 0.10 μV, *SE* = 0.13). There was no main effect of probe [*F* (5,145) = 0.84, *p* = 0.523], nor any matching degree × probe interaction [*F* (5,145) = 0.81, *p* = 0.548], possibly because differences between matched and mismatched stimuli were present across probes. To examine this, the amplitude of each probe was compared between match and mismatch conditions using a paired *t*-test. Figure 6c shows there were larger amplitudes elicited by target-matched stimuli than target-mismatched stimuli at probe 3 [*t* (29) = −1.81, *p* = 0.081, Cohen’s *d* = −0.33, 95% CI = [−0.70, 0.04], probe 4 [*t* (29) = −2.22, *p* = 0.034, Cohen’s *d* = −0.41, 95% CI = [−0.78, −0.03], probe 5 [*t* (29) = −2.69, *p* = 0.012, Cohen’s *d* = −0.49, 95% CI = [−0.87, −0.11], and probe 6 [*t* (29) = −2.50, *p* = 0.081, Cohen’s *d* = −0.46, 95% CI = [−0.83, −0.08]. The difference between probe 3 and probe 6 was only marginally significant.

In the strategy condition, a marginally significant main effect of matching degree was observed [*F* (1,29) = 3.53, *p* = 0.070, *η_p_*^2^ = 0.11], with larger amplitudes elicited by target-matched stimuli (*M* = −0.20 μV, *SE* = 0.16) than those elicited by target-mismatched stimuli (*M* = 0.14 μV, *SE* = 0.10). No significant main effect of probe was found [*F* (5,145) = 1.53, *p* = 0.184], but a significant interaction of matching × probe [*F* (5,145) = 2.46, *p* = 0.036, *η_p_*^2^ = 0.08] was present. Figure 7c shows larger amplitudes elicited by target-matched stimuli than those elicited by target-mismatched stimuli at probe 5 [*F* (1,29) = 5.78, *p* = 0.023, *η_p_*^2^ = 0.17] and probe 6 [*F* (1,29) = 8.69, *p* = 0.006, *η_p_*^2^ = 0.23]. For target-matched stimuli, N2pcs at probe 5 (*M* = −0.52 μV, *SE* = 0.24) and probe 6 (*M* = 0.73 μV, *SE* = 0.28) were larger than those at probe 1 (*M* = 0.09 μV, *SE* = 0.17, *p* = 0.026, *p* = 0.009), probe 3 (*M* = 0.12 μV, *SE* = 0.26, *p* = 0.064, FDR q-values = 0.126, *p* = 0.005), and probe 4 (*M* = −0.02 μV, *SE* = 0.26, *p* = 0.055, FDR q-values = 0.126, *p* = 0.005), with the differences between probe 5 and probe 3/4 being marginally significant. For target-mismatched stimuli, P_D_ at probe 4 (*M* = 0.51 μV, *SE* = 0.18) was larger than those at probe 1 (*M* = −0.33 μV, *SE* = 0.38, *p* = 0.069, FDR q-value = 0.126) and probe 2 (*M* = 0.06 μV, *SE* = 0.21, *p* = 0.050, FDR q-value = 0.126), with the difference between probe 4 and probe 1 being marginally significant.

The CAT effect was calculated by subtracting the amplitudes of target-mismatched stimuli from those of target-matched stimuli. To compare the CAT effect among the three category frameworks at each probe, the amplitudes were submitted to a one-way repeated-measures ANOVA with category framework (prototype, semantic, and strategy) as a factor. A significant main effect of category framework was found only at probe 6 [*F* (2,58) = 3.68, *p* = 0.038, *η_p_*^2^ = 0.21], showing that the CAT effect in the prototype condition (*M* = −1.75 μV, *SE* = 0.42) was larger than that in the semantic (*M* = −0.91 μV, *SE* = 0.36, *p* = 0.026) and strategy conditions (*M* = −0.90 μV, *SE* = 0.31, *p* = 0.030). No significant main effects were observed at probes 1–5 [*Fs* ≤ 2.10, *ps* ≥ 0.141].

**Figure 8:**
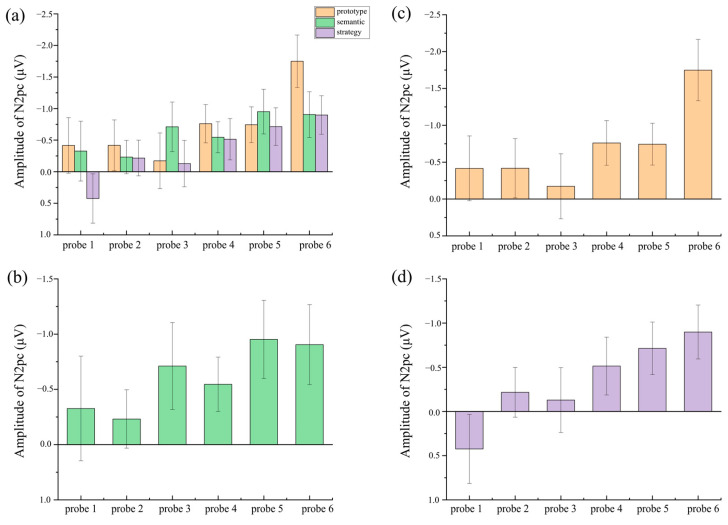
Different amplitudes of N2pcs (match minus mismatch) across probe 1–6 (**a**) in all three category conditions: (**b**) in the prototype category, (**c**) in the semantic category, (**d**) in the strategy category. The error bars indicate the standard errors.

To investigate the time course characteristics of CAT pre-activation for each category framework, separate one-way repeated-measures ANOVAs with probe (1–6) as a factor were conducted for the CAT effects. In the prototype condition, Figure 8b shows that the CAT effect at probe 6 (*M* = −1.75 μV, *SE* = 0.42) was significantly larger than at probe 1 (*M* = −0.42 μV, *SE* = 0.44, *p* = 0.009), probe 2 (*M* = −0.42 μV, *SE* = 0.40, *p* = 0.025), probe 3 (*M* = −0.17 μV, *SE* = 0.44, *p* = 0.011), probe 4 (*M* = −0.76 μV, *SE* = 0.30, *p* = 0.006), and probe 5 (*M* = −0.74 μV, *SE* = 0.28, *p* = 0.016). In the semantic condition, Figure 8c shows that the CAT effect at probe 5 (*M* = −0.95 μV, *SE* = 0.35) was marginally larger than at probe 2 (*M* = −0.23 μV, *SE* = 0.26, *p* = 0.053). In the strategy condition, Figure 8d shows that the CAT effect at probe 4 (*M* = −0.51 μV, *SE* = 0.33), probe 5 (*M* = −0.71 μV, *SE* = 0.30), and probe 6 (*M* = −0.90 μV, *SE* = 0.31) was significantly larger than at probe 1 (*M* = 0.42 μV, *SE* = 0.39, *p* = 0.031, *p* = 0.007, *p* = 0.001, respectively).

### 3.3. MVPA Results

**Figure 9:**
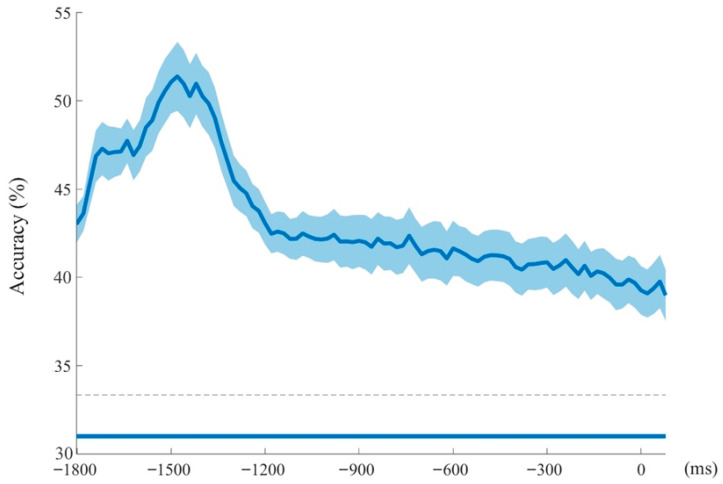
Classifier accuracy over time based on category. Colored bar on the x-axis indicates the time course of above-chance level decoding accuracy. The gray dashed line denotes the chance level.

To investigate the modulatory role of category on the time course of CAT pre-activation, the raw EEG signal of three category frameworks (prototype, semantic, and strategy) was decoded. Classification accuracy was tested against chance level (33.3%) using *t*-tests for each time point of the epoched data, with multiple comparisons corrected via 5000-iteration cluster-based permutation tests (*p* < 0.05). Figure 9 shows classification accuracy over time (−2000 ms to 100 ms) for whole brain activation. The blue bar above the x-axis indicates time periods with above-chance decoding, demonstrating distinct activation patterns among the three category frameworks. The highest classification accuracy, which exceeded 0.5, emerged at probe 2, beginning 50 ms before the onset of probe 2 and lasting until 100 ms after the onset of probe 2.

## 4. Discussion

By maintaining consistent task difficulty and balancing the target-defining dimensions, the current study investigated differences in the time course of CAT pre-activation across different category frameworks. The primary finding of this study was that the time course of CAT pre-activation can be modulated by category frameworks. Specifically, the semantic-based CAT was pre-activated earliest at about 1200 ms before the search display, followed by the prototype-based CAT at about 900 ms, with the strategy-based CAT being the latest at about 600 ms. By comparing different CAT frameworks, we found that the CAT represented by experience activation requires more preparation time. Relative to single features, CAT activation aligns more closely with how we search for objects in our daily lives, providing better guidance and enhancing the efficiency and accuracy of visual search tasks. The current study highlights the modulating effect of category framework during the preparation process before the target selection.

Our findings confirm the significant role of CAT in visual searching. CAT can be pre-activated to guide attention towards target-matched stimuli. During the activation phase, CAT allocates attention (N2pc), suppresses the distractors (P_D_), and changes the response performance (RTs and ACC). More importantly, we demonstrate that AT can extend to the category domains, facilitating the efficient processing of categorical-level information and adapting its mechanisms according to distinct CATs. Based on the ERPs and MVPA results of different activation modes of three CATs, we conclude that the semantic-based CAT is activated first in the pre-activation phase, indicating a greater requirement of preparation time compared to prototype-based and strategy-based CATs. The results of MVPA provided further evidence that the whole-brain pre-activation patterns of the three categories vary across probes. Notably, the decoding accuracy for the three categories peaked at probe 2, indicating that the three category frameworks can be distinguished at an early stage. Furthermore, there was a larger N2pc elicited by prototype and semantic conditions, indicating more attentional resource engagement for these two conditions in the pre-activation phase. However, during the activation phase at the search display, the ERP results showed that there was a smaller N2pc elicited by the semantic condition than that elicited by prototype and strategy conditions, indicating the competition between experience and learning in the pre-activation phase occupied attentional selection resources. Correspondingly, the semantic-based CAT also required longer reaction times in the response selection phase. Hence, our findings suggest that distinct CATs can exhibit different pre-activation and activation modes in visual search, and each of the CATs has its own special mode.

By examining the results of pre-activation, activation, and response selection, we observed differences in the activation mode of three CATs. The prototype-based CAT required less preparation time and demanded a larger engagement of attention resources throughout the whole search process. The prototype-based activation mode is determined by the characteristics of the prototype, which are involved in the similarity of the perceptual representation (Lech et al., 2016). As typical selections for the prototype-based category, the warm and cold colors are at the opposite ends of the spectrum, yielding a large discrepancy between categories and a large similarity within categories (Palmeri & Nosofsky, 2001). The discrepancy between target and distractor renders the target more salient to attract more attention, leading to a shorter response time and a larger amplitude of N2pc (Alexander & Zelinsky, 2012; Forschack et al., 2023; Yeh & Peelen, 2022). Meanwhile, relative to high-level conceptual information, the perceptual features abstracted from the prototype-based category are easier for individuals to quickly identify and lead to spending less time in the preparatory phase (Miao et al., 2023). Therefore, the prototype-based CAT elicited a relatively large N2pc (−1.85 μV) and demanded less preparation time (approximately 900 ms before the search display, at probe 4) during the pre-activation phase. The results relating to the time course are similar to previous research findings that reported the emergence of prototype-based CAT at 1000 ms before the search display (Grubert & Eimer, 2018). In the same way, due to the high perceptual similarity within the prototype-based category, the prototype-based CAT also showed a large strength of action in the activation phase, with a relatively larger N2pc (−2.22 μV). Based on these characteristics, the shorter reaction times and higher accuracy rate were observed in the response selection phase.

The semantic-based CAT required more preparation time and larger engagement of attention resources in the pre-activation phase, but a reduced investment in the activation phase. The semantic-based activation mode is influenced by the complexity representations of the semantic-based CAT, which require extracting content from working memory and matching it to the category-defining targets (Wu et al., 2020). This process required more time than the perception-dependent mode (Brockhoff et al., 2022; Tiferet-Dweck et al., 2016; Yurgil & Golob, 2013), and demanded greater attentional engagement (Wu et al., 2016). As a result, the semantic-based CAT required more preparation time and was pre-activated as early as approximately 1200 ms before the search display (at probe 3). Due to the complexity representations of the semantic-based CAT, it required greater attentional resources, resulting in a relatively larger N2pc (−1.79 μV) in the pre-activation phase. However, the whole activation process of CAT is successive; the excessive attentional resources occupied in the pre-activation phase result in insufficient resources in the activation phase, manifested as a relatively smaller N2pc (−1.31 μV). Another possible explanation is that the semantic-based CAT could be represented by experience and learning; when knowledge from learning is sufficient to facilitate the search, competition arises between experience and learning, ultimately negotiating a near-optimal search strategy (Brockmole & Le-Hoa Võ, 2010; Rehrig et al., 2021; Võ & Wolfe, 2012; Wynn et al., 2020). This competition and negotiation process consumes more time and requires more attentional resources in the pre-activation phase, leading to limited attentional resources in the activation phase. This competition of experience and learning also influenced the response selection phase, resulting in longer reaction times and a lower accuracy rate.

The strategy-based CAT required less preparation time and a smaller engagement of attention resources in the pre-activation phase, but a higher engagement in the activation phase. The strategy-based activation mode is determined by the definition of strategy-based CAT, which excludes the influence of experience. The strategy-based CAT is represented by learning rules; it is based solely on the current task and transient changes in task-related brain functional connections, which lead to reduced response time to complete tasks (Bueichekú et al., 2019). Because there are no experience factors, the strategy-based CAT does not require more preparation time and attentional resources to compete with experience (Rehrig et al., 2021; Võ & Wolfe, 2012). This allows more attentional resources to be allocated to the activation phase, thus optimizing performance and improving the search efficiency. Hence, the strategy-based CAT requires less preparation time (approximately 600 ms before the search display, at probe 5). The N2pc amplitude of the pre-activation phase was also smaller (−1.41 μV). Due to the adequate attentional resource, during the activation phase, the N2pc elicited by the strategy condition was relatively larger (−2.26 μV). Similarly, shorter reaction times and a higher accuracy rate were observed in the response selection phase. By comparing the results of the semantic-based CAT and the strategy-based CAT, we found that experience is the crucial factor that facilitates early pre-activation. According to the selective attention model (Broadbent, 1958), the allocation of attentional resources can be divided into an automated early selection stage and a refined late selection stage. Prior experience enables individuals to make predictions about the target automatically, allowing attention to be pre-guided to the target-matched cues about 1000 ms or more before the target actually appears (Grubert & Eimer, 2020; Wynn et al., 2020). Hence, the prior experience more likely plays an early screening role, influencing the early selection phase. In the current task, the learning factor was key, as utilizing knowledge of the stimuli enables precise guidance, optimizing the allocation of attentional resources when the search task approaches (Broadbent, 1958; Grubert & Eimer, 2020). Hence, the learning process is more likely to operate on the later stages of pre-activation. Overall, the present results indicate that experience can impact the early time course of pre-activation.

Overall, our results revealed distinct activation modes across the three category frameworks, suggesting that different CATs engage separable neural mechanisms during pre-activation and activation. From another perspective, however, it is worth noting that both the prototype-based and strategy-based categories were linearly separable in the perceptual color space, raising the possibility that their activation differences might, to some extent, be related to perceptual discriminability. Nevertheless, although the colors in the prototype- and strategy-based category frameworks were both linearly distributed, their pre-activation patterns showed significant differences; the prototype-based CAT exhibited earlier (~900 ms before target onset) and stronger pre-activation, reflecting greater attentional engagement, whereas the strategy-based CAT showed later (~600 ms before target onset) and weaker pre-activation, reflecting more transient preparatory processing. The prototype-based CAT required longer preparation and engaged stronger attentional allocation across both pre-activation and activation phases, whereas the strategy-based CAT showed a more transient preparatory process followed by enhanced activation at the search display. These contrasting patterns indicate that, despite both category frameworks having linearly distributed colors, the pre-activation dynamics are primarily determined by the categorical structure rather than by color distribution. To support this interpretation, we conducted additional statistical analyses comparing the ERP responses for each typical color (red, blue, yellow, and green) across the three category frameworks (see Supplementary Figure S1). The results showed no significant main effects or interactions for any of the individual colors, confirming that differences in activation patterns are not driven by perceptual discriminability but by category-specific mechanisms.

Interestingly, the reliable P_D_s were elicited by target-mismatched stimuli in the prototype and strategy conditions, indicating that the non-target stimuli could be inhibited when dealing with a complex visual search task. The signal suppression hypothesis posits that the singleton distractors can be inhibited by a top-down process and can elicit significant P_D_ components, which can explain the current results showing that stimuli can be pre-emptively suppressed to prevent distraction when they are task-irrelevant (Drisdelle & Eimer, 2021; Gaspelin & Luck, 2018a, 2018b; Sawaki & Luck, 2010). In contrast, consistent with the findings of Miao (Miao et al., 2023), no reliable P_D_ components were elicited under the semantic-based category. One possible explanation for this is that semantic-based categories involve complex concepts that are more susceptible to interference and are more easily ignored. Maintaining such complex information in working memory may demand greater attentional resources, leaving fewer resources available for distractor suppression (Brockhoff et al., 2022; Oberauer & Kliegl, 2001; Thibaut et al., 2010). Another possibility, supported by recent findings (Chen et al., 2023; Zhang et al., 2024), is that the amplitudes of N2pc and P_D_ components are influenced by the saliency of stimuli. In this context, the same colors might have different mental saliency under distinct category frameworks, which could result in varying N2pc and P_D_ responses and differences in their pre-activation timing. Future research could further explore how information complexity and mental saliency affect distractor suppression.

## 5. Conclusions

In summary, this study provided ERP and MVPA evidence for different pre-activation patterns among category frameworks under conditions that balanced both the task difficulties and target dimensions. Importantly, the semantic-based CAT was elicited earlier than prototype-based and strategy-based CATs, indicating that the influence of experience contributed to an early time course of pre-activation. This study extends our understanding of the neural basis of category information decoding and attentional resource allocation, not only providing significant insights into the pre-activation mechanisms of CAT, but also offering practical guidance for optimizing attention control in real-world applications.

## Data Availability

All the data and code necessary to reproduce the experiment and analyses are available online at https://osf.io/y7ujd/ (accessed on 20 June 2025).

## Supplementary Materials

The following supporting information can be downloaded at https://www.mdpi.com/article/10.3390/bs15121606/s1. Figure S1: ERP results in the prototype, semantic, and strategy conditions.
