# Supplementary material for: Pre-Activation Dynamics of Category-Specific Attentional Templates in Visual Search
Source: Behav Sci (Basel). 2025 Nov 21;15(12):1606. doi: 10.3390/bs15121606 (PMC12729359; doi:10.3390/bs15121606)
Supplement: Supplementary file 1 [file behavsci-15-01606-s001.zip › behavsci-3927309-supplementary.pptx]

## Slide 1
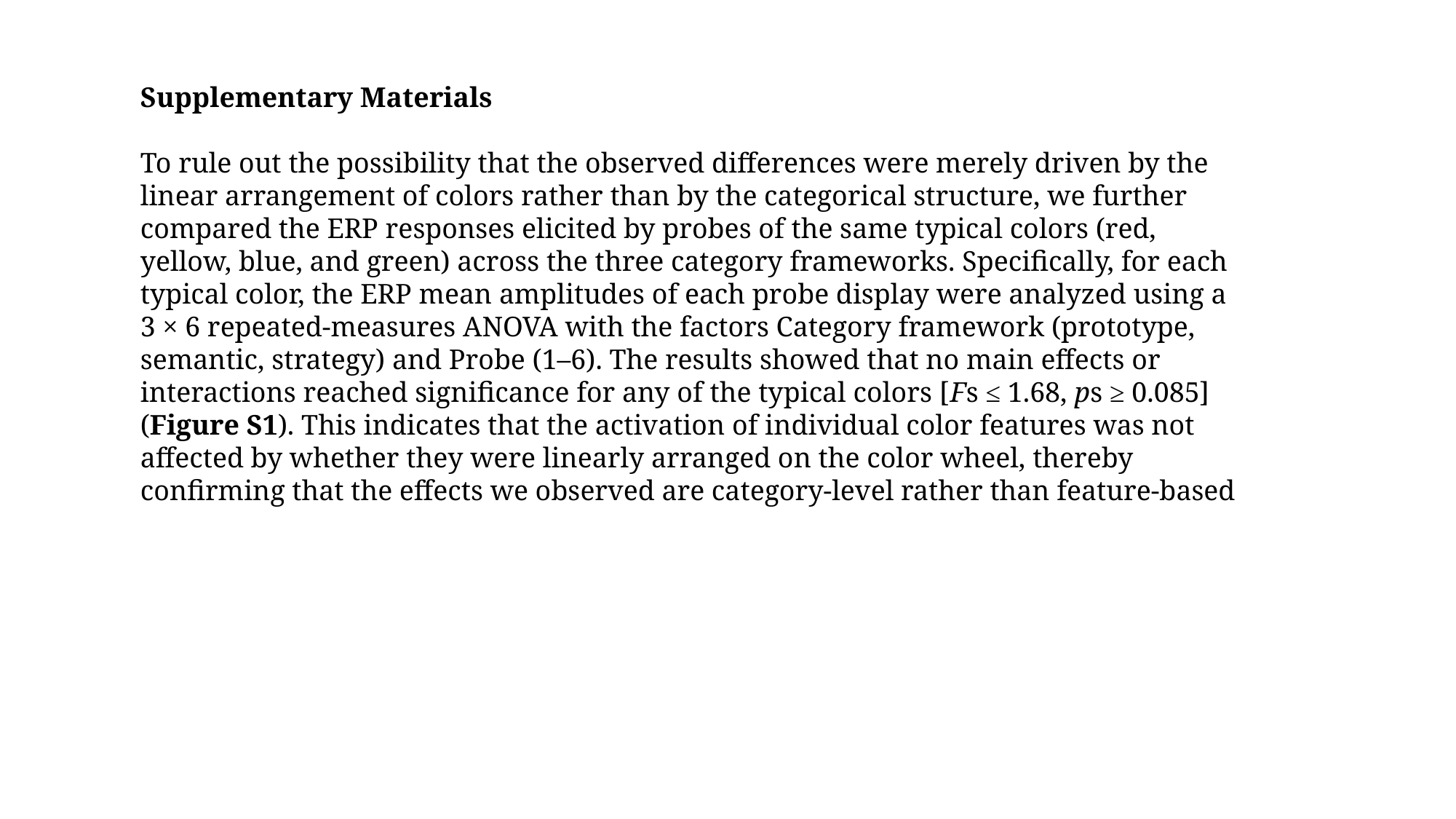

Supplementary Materials
To rule out the possibility that the observed differences were merely driven by the linear arrangement of colors rather than by the categorical structure, we further compared the ERP responses elicited by probes of the same typical colors (red, yellow, blue, and green) across the three category frameworks. Specifically, for each typical color, the ERP mean amplitudes of each probe display were analyzed using a 3 × 6 repeated-measures ANOVA with the factors Category framework (prototype, semantic, strategy) and Probe (1–6). The results showed that no main effects or interactions reached significance for any of the typical colors [Fs ≤ 1.68, ps ≥ 0.085] (Figure S1). This indicates that the activation of individual color features was not affected by whether they were linearly arranged on the color wheel, thereby confirming that the effects we observed are category-level rather than feature-based

## Slide 2
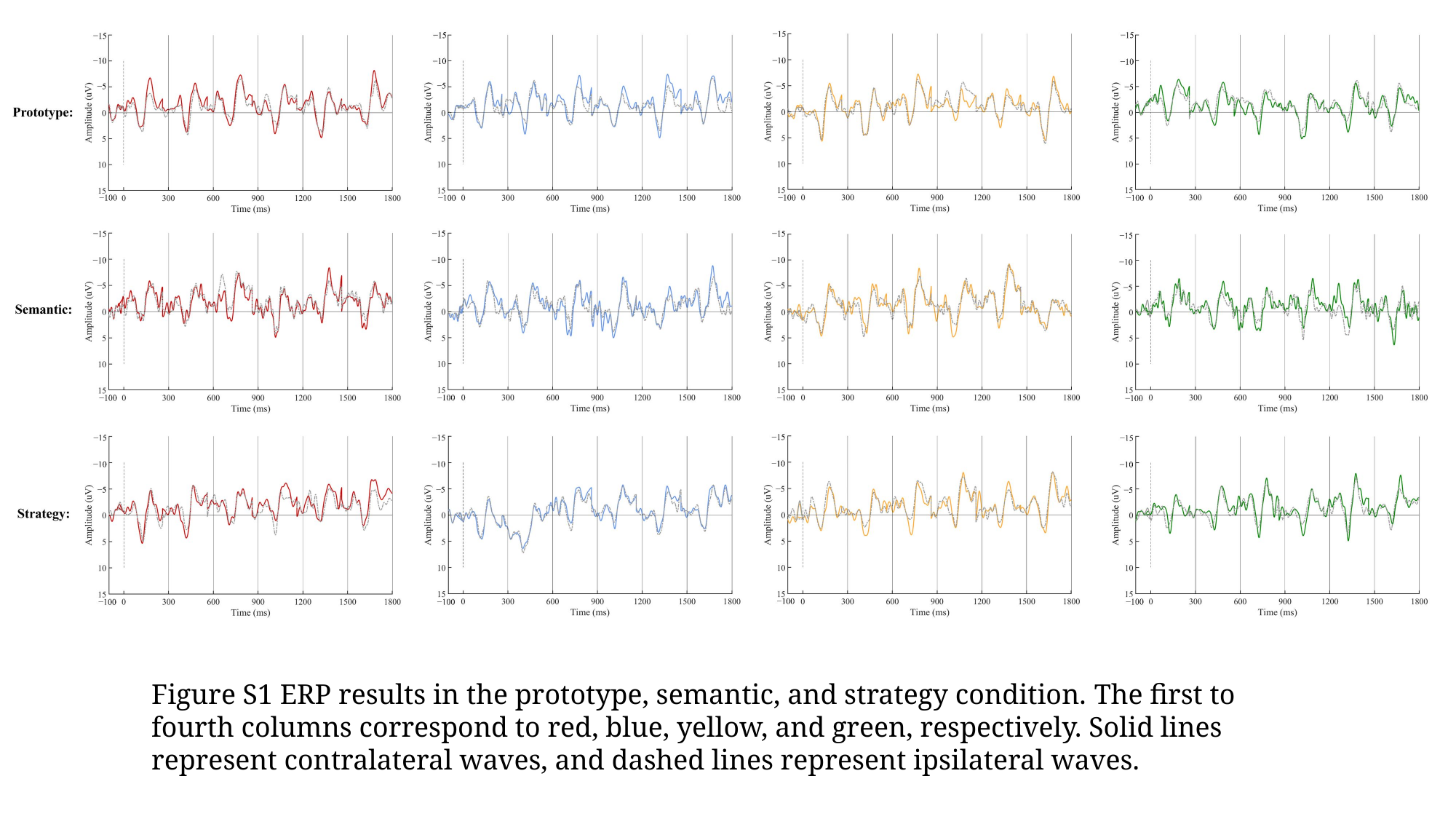

Figure S1 ERP results in the prototype, semantic, and strategy condition. The first to fourth columns correspond to red, blue, yellow, and green, respectively. Solid lines represent contralateral waves, and dashed lines represent ipsilateral waves.
